# Orthotopic Liver Transplantation After Stereotactic Body Radiotherapy for Pediatric Hepatocellular Carcinoma with Central Biliary Obstruction and Nodal Involvement

**DOI:** 10.7759/cureus.3499

**Published:** 2018-10-26

**Authors:** Emily Chen, Arun Rangaswami, Carlos O Esquivel, Waldo Concepcion, Matt Lungren, Avnesh S Thakor, Christopher H Yoo, Sarah S Donaldson, Susan M Hiniker

**Affiliations:** 1 Radiation Oncology, Stanford University School of Medicine, Stanford, USA; 2 Pediatric Hematology / Oncology, Lucile Packard Children's Hospital, Stanford, USA; 3 Surgery, Stanford University School of Medicine, Stanford, USA; 4 Surgery, Lucile Packard Children's Hospital, Stanford, USA; 5 Interventional Radiology, Stanford University School of Medicine, Stanford, USA; 6 Radiation Oncology, Stanford University Medical Center, Stanford, USA

**Keywords:** transplant, downstaging, sbrt, hepatocellular carcinomas (hcc), pediatric oncology

## Abstract

Here we describe the case of a 10-year-old boy with a history of chronic hepatitis B who was diagnosed with hepatocellular carcinoma (HCC) with a large central hepatic mass and metastatic disease in a celiac lymph node. His tumor was unresectable, due to location and lack of clear margins, and he could not receive chemotherapy due to elevated bilirubin. He was treated with stereotactic body radiotherapy (SBRT) to the primary site and involved nodal region. After completing radiotherapy, his total bilirubin level fell below 1.0 mg/dL, allowing him to begin systemic therapy with cisplatin and doxorubicin. At three months after SBRT, his bilirubin was 0.1 mg/dL, alpha-fetoprotein (AFP) was 88 ng/mL, and imaging demonstrated a decrease in tumor size (total volume 28.7 cc), with no evidence of local or distant disease progression. He then developed distant disease within the liver, but his disease remained controlled at the primary site and nodes that had been treated with SBRT. He underwent orthotopic liver transplantation (OLT) with an uneventful operative course and remains with no evidence of disease at seven months after OLT. This is one of the first reported cases of successful downstaging of pediatric HCC with nodal involvement to allow for OLT, and it argues for consideration of similar patients for OLT.

## Introduction

Hepatocellular carcinoma (HCC) is the second most common primary liver tumor among children. The primary curative treatment for patients is surgical resection, and the only curative approach for children with unresectable disease is orthotopic liver transplantation (OLT). However, patients with nodal disease are frequently not considered candidates for OLT.

We report the case of a 10-year-old boy with a history of chronic hepatitis B who was diagnosed with hepatocellular carcinoma with an unresectable central hepatic mass and involvement of a celiac lymph node. He was treated with stereotactic body radiotherapy (SBRT) to the primary site and celiac nodal region with excellent response in bilirubin allowing him to begin systemic therapy. He ultimately experienced distant progression within the liver and given the lack of evidence of active disease outside the liver, he was treated with OLT.

## Case presentation

**Figure 1:**
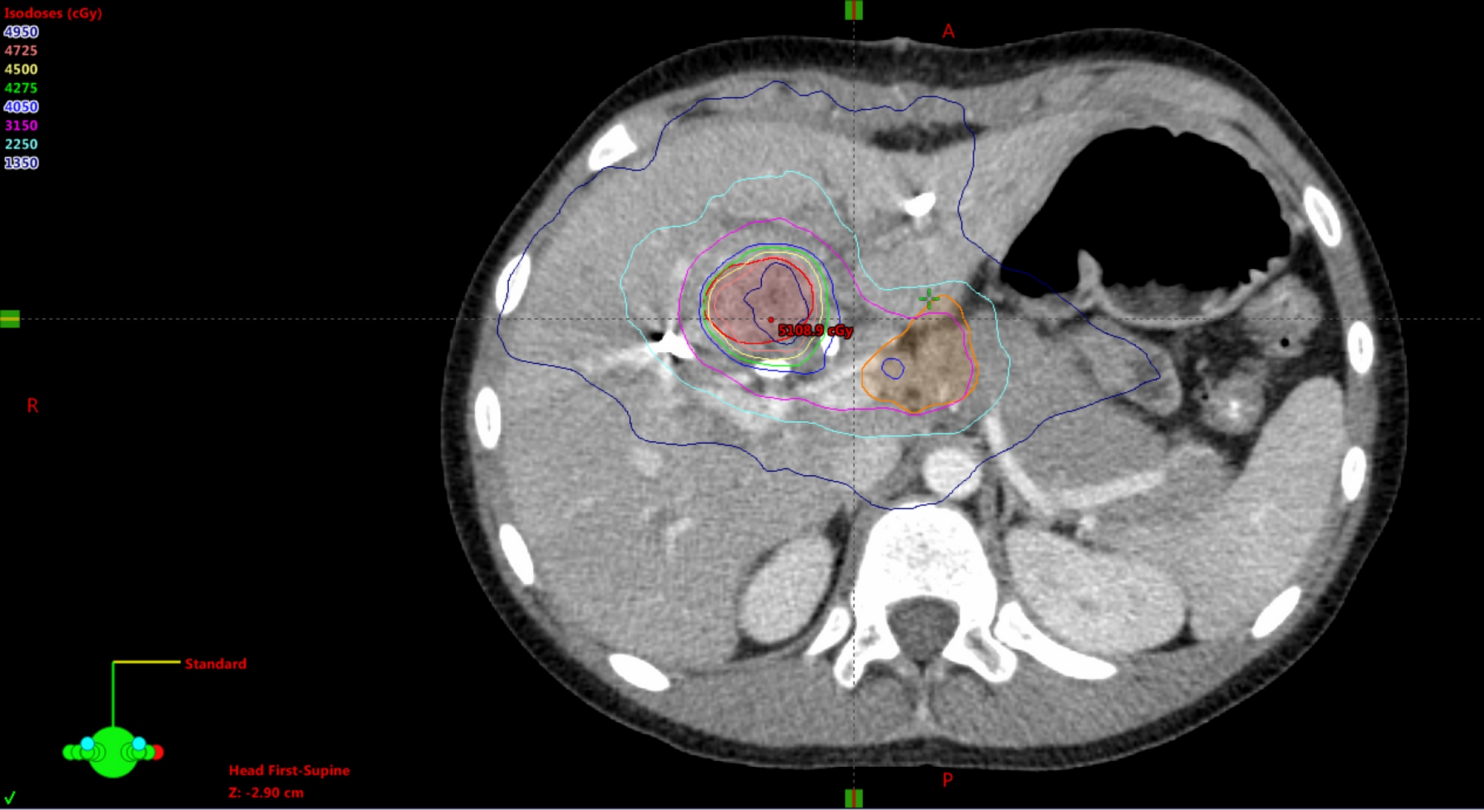
Stereotactic body radiotherapy treatment plan for central hepatocellular carcinoma with nodal involvement

In our initial case report [1], we described the case of a 10-year-old boy with a history of chronic hepatitis B, whose parents also had chronic hepatitis B, who was diagnosed with hepatocellular carcinoma (HCC) with a central hepatic mass 43.5 cc in volume. At diagnosis, he had metastatic disease in a celiac lymph node. His tumor was deemed unresectable due to the location and lack of clear margins, while he was also not a candidate for liver transplantation, doxorubicin-containing systemic chemotherapy, radiofrequency ablation, radioembolization, or transarterial chemoembolization. Given the paucity of therapeutic options, he was treated with stereotactic body radiotherapy (SBRT), with his primary site receiving 45 Gy in five fractions and the celiac nodal region receiving 35 Gy in five fractions (Figure 1). After completing radiotherapy, his total bilirubin level fell below 1.0 mg/dL, allowing him to begin systemic therapy following the SIOPEL4 Block A2 regimen with cisplatin 70 mg/m2 and doxorubicin 30 mg/m2 [2] which he received for one cycle. Our previous report concluded at three months follow-up, when his bilirubin was 0.1 mg/dL, alpha-fetoprotein (AFP) was 88 ng/mL, and imaging demonstrated a decrease in tumor size (total volume 28.7 cc), with no evidence of local or distant disease progression.

Repeat magnetic resonance imaging (MRI) of the abdomen performed four months after completion of SBRT showed a further decrease in the size of the central hepatic mass, now measuring 2.8 x 2.4 cm compared to the previous measurement of 3.8 x 3.5 cm. Repeat imaging performed six and seven months after completion of radiation continued to show mild decrease in the size of the mass to a size of 2.2 x 1.8 cm.

However, 8.5 months after completion of SBRT, an abdominal MRI demonstrated the central hepatic mass stable in size but also showed an enlarging secondary hepatic lesion (1.3 x 1.2 cm, previously 0.7 x 1.0 cm) as well as new additional scattered lesions in the right peripheral lobe of the liver, which measured 1.0 cm, consistent with disease progression. Due to the prior SBRT, he was not a candidate for radioembolization. The liver transplant committee reviewed his case but deferred listing him for liver transplantation because he had presented with extrahepatic disease and thus failed to meet the United Network for Organ Sharing (UNOS) criteria for transplantation and also due to concern that the original tumor may have contained an element of cholangiocarcinoma, thus portending a poorer prognosis [3]. The committee agreed to reconsider the option of transplantation if repeat biopsy failed to show cholangiocarcinoma and positron emission tomography (PET) imaging did not detect metastatic disease.

The patient underwent interventional radiologic (IR)-guided biopsy of the hepatic lesions, but pathology was inconclusive as to whether the process represented HCC or cholangiocarcinoma. Therefore, he was not considered a candidate for hepatic transplantation. However, the PET imaging demonstrated non-avidity, favoring a diagnosis of HCC. He subsequently began systemic chemotherapy with an individualized protocol of gemcitabine 1000 mg/m2 and oxaliplatin 85 mg/m2. Repeat MRI abdominal imaging following two cycles of chemotherapy demonstrated interval growth of the previously noted smaller hepatic lesions, as well as appearance of a new lesion within the left hepatic lobe, though notably the original SBRT-treated lesion did not grow. Given imaging findings suggestive of disease progression, gemcitabine/oxaliplatin was discontinued and the patient was switched to systemic therapy with vincristine 2 mg, irinotecan 50 mg/m2, and temsirolimus 35 mg/m2 (VIT), as per AHEP0731, Regimen H [4].

Repeat abdominal imaging after cycles two, four, and six of VIT demonstrated stable disease. AFP levels also declined throughout treatment (51 ng/ml post-cycle two, 37 ng/ml post-cycle four, 38 ng/ml post-cycle six). The liver transplant committee was consulted again after the patient completed cycle eight of VIT and decided to move forward with a laparoscopic biopsy of a suspicious portocaval lymph node to determine if there was any active nodal disease present. The biopsy showed no sign of carcinoma and the patient was thus listed for consideration of hepatic transplantation with a Pediatric End-Stage Liver Disease (PELD) score of 40.

Two weeks later, the patient underwent orthotopic liver transplant (OLT) and had an uneventful operative course. However, his transaminase levels rose acutely during the first several postoperative days (POD); thus, he underwent liver biopsy on POD nine, which failed to confirm rejection and was more consistent with preservation injury, defined as hepatic dysfunction caused by cold or warm ischemia. His postoperative course was further complicated by influenza B infection and significant acute kidney injury (AKI) secondary to nephrotoxic medications of vancomycin and tacrolimus.

Over the following two months, he completed cycles nine and ten of VIT. Follow-up imaging performed two months after OLT showed evidence of complete remission. He remains with no evidence of disease seven months after OLT. He continues on tenofovir with hepatitis B DNA undetectable by polymerase chain reaction (PCR).

## Discussion

HCC is the second most common primary hepatic malignancy of children aged five to 10 years [5]. HCC occurs with two incidence peaks: the first during the early ages of 15-25 years, accounting for 80% of liver tumors in this age group, and the second peak occurring between ages 55 and 64 years [6]. HCCs diagnosed during the first age range represent a clinical and biologically heterogeneous group of tumors that most often develop in healthy hepatic tissue and are associated with etiological factors that are unknown or different from those appearing in adults, with the exception of tumors associated with hepatitis B virus (HBV) infection.

There is evidence that pediatric HCC is more chemoresponsive than HCC presenting in adults [7]. Thus, for children with resectable disease, the primary treatment options center on surgical resection and systemic chemotherapy. Given the poor survival rate without resection, OLT remains the only curative option for children with unresectable but localized disease. The outcome for patients presenting with locoregional or distant metastases is very poor [2].

The benchmark that has guided candidacy for OLT for adults over the last 20 years has been the Milan criteria (one lesion ≤5 cm or two to three lesions ≤3 cm) or the University of California, San Francisco (UCSF) criteria (single tumor <6.5 cm, maximum of three total tumors with none >4.5 cm, and cumulative tumor size <8 cm) [8, 9]. However, debate exists whether these criteria for adults should apply to the pediatric and young adult population, as some consider them to be too restrictive. Many clinicians who care for children and young adults propose expanding these criteria [10]. Amid this debate, an active area of research is the investigation of successful methods of “down-staging” or “bridging,” as a means to reducing tumor burden for those previously considered ineligible by the Milan/UCSF criteria for transplantation via locoregional disease therapy [11]. Studies that have investigated patient outcome following OLT after down-staging have demonstrated the efficacy of this approach [11, 12].

We believe that the Milan/UCSF eligibility criteria for adults undergoing OLT should not be applied to all pediatric HCC patients; rather, we believe each case should be individually assessed for consideration of transplantation. Our case demonstrates that the principle and practice of down-staging may be relevant to children and adolescents given how the eligibility criteria would have precluded our patient from consideration for both OLT and even down-staging using locoregional approaches due to his extrahepatic disease at the time of diagnosis. We have shown that SBRT and systemic chemotherapy can be used successfully to reduce tumor burden to a level acceptable for OLT; our patient remains well following transplantation.

This case shows the efficacy of SBRT in the multimodal management of pediatric HCC, particularly in the setting of a tumor causing local obstruction precluding other treatment modalities. HCC is known to be a moderately radiosensitive tumor, and as a parallel organ, small volumes of the liver can be treated with a high dose of radiation with a relatively low risk of damage. However, we caution that outcome of similarly treated patients must be reported in order to advance the evidence for this approach. Recently, an international trial studying the management of hepatocellular neoplasms, the Pediatric Hepatic International Tumor trial (PHITT/ COG AHEP1531) has been opened in North America, Europe, and Japan [13, 14]. The arm of this trial for unresectable disease utilizes locoregional approaches in a prospective fashion in order to down-stage patients and render them either resectable or eligible for OLT.

## Conclusions

To our knowledge, this is the first report of successful down-staging of a pediatric case of HCC with nodal disease treatment followed by OLT. Others have shown that clinical outcome for pediatric HCC patients who undergo OLT is superior to those treated with conventional means via resection and chemotherapy, regardless of prior treatment history and fulfillment of adult Milan criteria. Together, this argues for further investigation into methods for facilitating the attainment of OLT eligibility for pediatric HCC patients.
